# Tumeur de la muqueuse buccale

**DOI:** 10.11604/pamj.2014.18.113.4612

**Published:** 2014-06-04

**Authors:** Iman Hadj, Fatima Zahra Mernissi

**Affiliations:** 1 CHU Hassan II, Fès, Maroc

**Keywords:** Diapneusie, muqueuse buccale, tumeur, diapneusia, buccal mucosa, tumor

## Image en medicine

La diapneusie est une tumeur bénigne correspondant à une hyperplasie fibro-épithéliale, Siégeant sur la muqueuse des lèvres, des joues ou des bords de la langue. Elle est attribuée à l'aspiration de la muqueuse à travers un orifice de l'arcade dentaire. Cliniquement, elle se présente sous forme d'un nodule sessile de quelques millimètres de diamètre, de consistance molle, parfois ferme, indolore, souple, sa surface est recouverte d'une muqueuse normale et le sommet de la lésion est parfois occupé par une plage kératosique ou une ulcération associée au traumatisme masticatoire. Elle est toujours bénigne mais elle récidive tant que l’étiologie n'a pas été éliminée. Le traitement consisté en l'exérèse chirurgical, enseignement pour éviter le tic de succion,et le traitement orthodontique pour fermer les espaces et corriger le chevauchement qui est en cause du tic de succion. Nous rapportons le cas d'une patiente de 19 ans, sans antécédents pathologiques notables, qui consulte pour une tumeur de la face interne de la joue droite évoluant depuis 3 mois. L'examen clinique révèle la présence d'une tumeur sessile de 15 millimètres de diamètre, de consistance ferme, indolore, souple,la muqueuse de recouvrement est d'aspect normal avec au sommet une plage kératosique.la patiente a bénéficié d'une exérèse chirurgicale avec étude anatomopathologique qui a confirmé le diagnostic en montrant un tissu de collagène quasi avasculaire, emprisonnant des fibres musculaires désorganisées recouvert d'un épithélium malpighien hyperplasique, légèrement kératinisé en surface avec absence de tissu adipeux et de réaction cellulaire inflammatoire.

**Figure 1:**
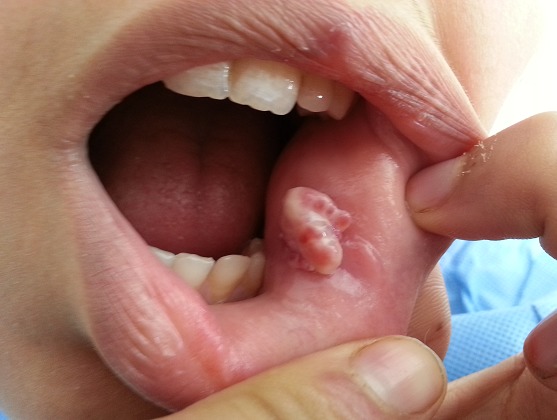
Tumeur sessile de 15 millimètres de diamètre, de consistance ferme, indolore, souple. La muqueuse de recouvrement est d'aspect normal avec au sommet une plage kératosique

